# Identifying Cost-Burdened Older Adults: What Is the Best Measure?

**DOI:** 10.1093/geroni/igaa057.2416

**Published:** 2020-12-16

**Authors:** Samara Scheckler, Sarah Mawhorter, Jennifer Molinsky, Alex Hermann, Whitney Airgood-Obrycki

**Affiliations:** 1 Harvard University, Cambridge, Massachusetts, United States; 2 University of Southern California, Syracuse, New York, United States

## Abstract

While affordable housing typically describes housing costs that fall within 30 percent of total income, older adult spending systematically differs from younger cohorts. For instance, budgets may skew away from mortgages and towards home modifications while medical or personal care expenses can drive monthly costs. This research uses the Health and Retirement Study (HRS) to explore various older adult housing cost burden measures and identify their relative advantages. Measures of cost burden are applied to HRS respondents and different cost burdened groups are defined. The welfare of each group is then assessed using metrics such as unmet need and caregiver stress. Findings suggest that traditional cost burden measures identify many vulnerable older adults. However, other measures of cost burden can highlight older adults who are disproportionately impacted by medical cost. This research should help professionals better align the metric that defines their target population members with their policy area.

